# Data on pteridophyte species diversity and status of the International Union for Conservation of Nature in each political unit of Myanmar

**DOI:** 10.1016/j.dib.2020.105503

**Published:** 2020-04-20

**Authors:** Phyo Kay Khine, Harald Schneider

**Affiliations:** Macroevolution Group, Center for Integrative Conservation, Xishuangbanna Tropical Botanical Garden, Chinese Academy of Sciences, China

**Keywords:** Conservation status, Ferns and lycophytes, Myanmar, Political unit, Pteridophytes, Species diversity, Species list

## Abstract

The data in this article provide information about the species richness of each political unit in Myanmar and their preliminary conservation status. The dataset was compiled by gathering scattered data from different sources. The majority of the data were compiled from the authors’ collections and historical and recent floristic treatments. Online databases and herbarium specimens were additional resources used for data compilation. In total, 603 species were documented and species names were standardized in accordance with the International Plant Names Index and Pteridophyte Phylogeny Group. Species composition between political units was calculated using the minimum variance of the Jaccard's dissimilarity matrix. Conservation status for 558 pteridophyte species from Myanmar was evaluated based on the global occurrences and occurrences only in Myanmar, and species with less than three occurrences were excluded from the analysis. This dataset can be applied as the reference data for future taxonomic revisions, biodiversity assessments, and conservation planning. Moreover, the data can be used for global diversity assessment, broad-scale species distribution modeling and bioclimatic modeling. The data provided in this manuscript have been analyzed and discussed in Khine and Schneider [1].

**Table:** Specifications table

Subject	Plant Science
Specific subject area	Biodiversity and conservation
Type of data	Table Figure
How data were acquired	The data were compiled from several sources including authors’ collections, historical and current publications, herbarium records, and online databases.
Data format	Raw Analyzed
Parameters for data collection	Pteridophytes species occurred in each political unit; species composition among political units; extent of occurrence; Area of occupancy; conservation status
Description of data collection	A large part the dataset was obtained from authors’ collections in northern and northwestern Myanmar between 2012 and 2014, and recent and historical publications. Additionally, species occurrence records accessible via Global Biodiversity Information Facility (GBIF) were included. The missing localities of species occurrences from online databases were traced in the relevant literature and identified using specimens from herbaria where most of the Myanmar pteridophytes’ collections were deposited such as Natural History Museum, London, New York Botanical Garden and Naturalis Biodiversity Center.
Data source location	Myanmar 8°N and 29°N, and 92°E and 102°E
Data accessibility	With the article, Direct URL to data: https://data.mendeley.com/datasets/zvrymn7r8z/1
Related research article	Phyo Kay Khine, Harald Schneider, First assessment of pteridophytes’ composition and conservation status in Myanmar, Global Ecology and Conservation. https://doi.org/10.1016/j.gecco.2020.e00995

## Value of the data

• The dataset provides the pteridophyte conservation status, diversity, and composition of a species-rich but under-sampled region in Southeast Asia. Moreover, it fills the data gap and contributes to our better understanding of pteridophyte diversity and distribution in East and Southeast Asia.
• The dataset can provide information for researchers who focus on global floristic assessment, bioclimatic modeling and biodiversity conservation.
• This first dataset on species diversity of each political unit highlights the priority regions and diversity hotspots, and can be used as a baseline for management and conservation planning.
• This dataset gives insight into the rarity and commonness of species recorded in Myanmar and can be a reference list for taxonomic revision in Myanmar, as pteridophyte taxonomy has not been updated since 1946.

## 1 Data description

**Fig. 1:**
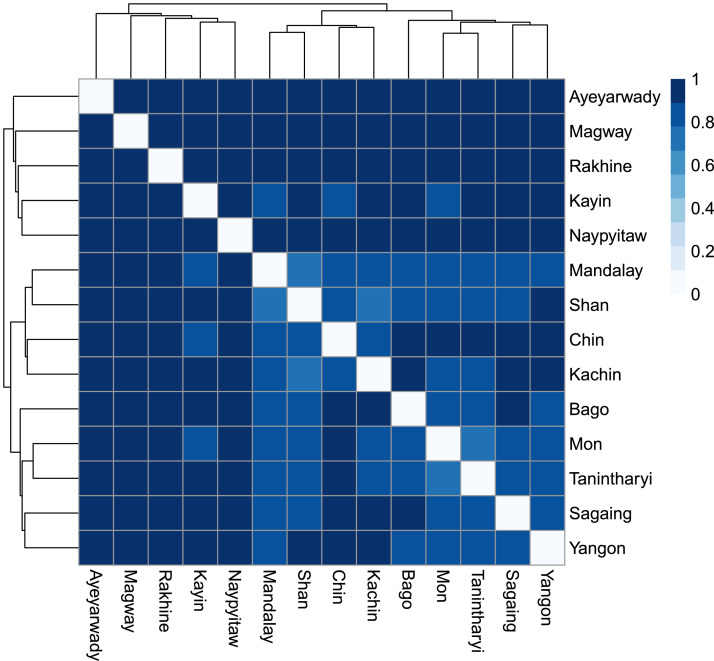
Visualization of the species dissimilarity among the political units in Myanmar. The values close to zero represent high similarity whereas the values close to 1 correspond to low similarity.

The dataset attached to this manuscript provides information about pteridophyte species in fourteen political units (Ayeyarwady Region, Bago Region, Chin State, Kachin State, Kayin State, Mandalay Region, Magway Region, Mon State, Naypyitaw Union Territory, Rakhine State, Sagaing Region, Shan State, Tanintharyi Region, and Yangon Region) in Myanmar, one of the most under-sampled regions in Southeast Asia. The dataset includes the list of all pteridophyte species recorded in Myanmar (Table 1), 558 species of which have been evaluated for conservation status (Table 2). The visual orientation of species composition between the political units is displayed in Fig. 1.

Fig. 1 presents the hierarchical clustering of the Jaccard dissimilarity based on minimum variance in fourteen political units in Myanmar. The first cluster in the dendrogram consists of the political units with distinct species, whereas the other two clusters include the political units with similar species. The values in the figure range from 0 (similar, white) and 1 (dissimilar, dark blue). **Table 1** presents the list of 603 pteridophyte species describing the presence (=1) and absence (=0) of individual species in each political unit of Myanmar. Kachin State (389 species) is the most species-rich region in Myanmar followed by Shan State (181) and Chin State (127). **Table 2** includes 558 species which have above three occurrences in global distribution and presents the unique occurrence of taxa in each political unit, extent of occurrence globally (EOO) and in Myanmar (EOO_MM), area of occupancy globally (AOO) and in Myanmar (AOO_MM), and preliminary global conservation status for pteridophyte species recorded in Myanmar based on the IUCN criterion B1 and B2. EOO of less than 100 km^2^ and AOO of less than 10 km^2^, less than 5000 km^2^ and 500 km^2^, less than 20,000 km^2^ and 2,000km^2^, and more than 20,000 km^2^ and 2,000km^2^ were assigned as Critically Endangered (CR), Endangered (EN), Vulnerable (VU), and Near Threatened or Least Concern (NT or LC), respectively [2]. The Critically Endangered (CR) category is not evaluated as the species with less than three occurrences are excluded.

**Table 1** Presence of species in each political unit of Myanmar are listed for all 603 pteridophyte species.

Direct URL to data: https://data.mendeley.com/datasets/zvrymn7r8z/1

**Table 2** Preliminary IUCN status assessment for Criteria-B (B1 and B2) for 558 pteridophyte species in Myanmar. Number of unique occurrences in Myanmar, extent of occurrence (EOO for global occurrence, EOO_MM for occurrence in Myanmar), Area of occupancy (AOO for global occupancy and AOO_MM for ocupancy in Myanmar) and preliminary conservation status are listed in the table.

Direct URL to data: https://data.mendeley.com/datasets/zvrymn7r8z/1

## 2 Experimental design, materials, and methods

### 2.1 Compilation of pteridophyte species dataset

We compiled a list for pteridophyte species richness of each political unit of Myanmar (8°N and 29°N, and 92°E and 102°E), namely Ayeyarwady Region, Bago Region, Chin State, Kachin State, Kayin State, Mandalay Region, Magway Region, Mon State, Naypyitaw Union Territory, Rakhine State, Sagaing Region, Shan State, Tanintharyi Region, and Yangon Region. Several data sources were used to obtain this information including (1) field data collections obtained by the authors, and (2) historical records as accessible via online searches of herbaria databases supported by GBIF (https://www.gbif.org/) or direct searches of the herbarium databases of the Natural History Museum, London (http://www.nhm.ac.uk/), New York Botanical Garden (https://www.nybg.org/), and Naturalis Biodiversity Center (https://science.naturalis.nl/). Between 2012 and 2014, a total of 299 species were reported from north and northwestern Myanmar [3], 125 of which were newly added to the previous checklist of Dickason [4] in which 460 species were listed. Additional species were acquired from the online database and thus the current dataset includes 603 pteridophyte species. The major challenges for compiling the data from several sources, especially for a country with a lack of pteridophyte revision since 1946, were taxonomic inconsistency and missing localities. Moreover, the name of many localities in Myanmar has changed during the last century, providing a major obstacle to assigning each species to each political unit. The missing locality names were pursued in every searched herbarium specimen and relevant literature and traced based on the collection routes of American and European naturalists and plant hunters who conducted their research in Myanmar in the late 19th and early 20th century. To reduce transcription errors, we followed the nomenclature of the Pteridophyte Phylogeny Group [5] and standardized the species name in accordance with the International Plant Names Index (https://www.ipni.org/). We also took into account the specific recommendations of the “Shenzhen Code” of International Code of Nomenclature for Algae, Fungi, and Plants (https://www.iapt-taxon.org/nomen/main.php).

### 2.2 Assessment of species composition and conservation status

Similarities and differences in species composition between political units were explored by calculating pairwise comparison of species sets. The Jaccard index for presence/absence data can be defined by weighing of the shared species in both units by the sum of the species occurring in each unit and shared species in both units [6]. The “vegdist” function in “vegan” package was applied using the “jaccard” method to calculate the community dissimilarity. Hierarchical clustering of the political units was analyzed based on the minimum variance using a previously produced dissimilarity matrix [7]. The “hclust” function with the “ward.D” method, and “pheatmap” and “RColorBrewer” functions, were applied to produce the dendrogram and heatmap, respectively.

To avoid the overestimation of the threatened categories for pteridophyte species in Myanmar, two approaches to access the IUCN conservation status were applied. The first assessment for the species recorded in each political unit was carried out using exclusively occurrences of those species in Myanmar. Based on the extent of occurrence and area of occupancy, most of the species were assigned to the threatened categories. However, two third of those species were not globally threatened, therefore, the finding promoted the overestimation of conservation concern in an under-sampled region. The second assessment included the global occurrences of species recorded in Myanmar as accessed in March 2019 via GBIF. Species having less than three occurrences globally were excluded from the assessment, therefore, 558 species were evaluated for the IUCN criteria, B1 and B2. The conservation status for both evaluations was calculated using the “IUCN.eval” function in “ConR” package. We used the R statistical platform to perform all analyses and produce the figure [8]. For more detailed interpretations and discussions, refer to Khine & Schneider's publication [1].
